# Effectiveness of Aerobic, Resistance, and Combined Training for Hypertensive Patients: A Randomized Controlled Trial

**DOI:** 10.4314/ejhs.v33i6.17

**Published:** 2023-11

**Authors:** Addis Alemayehu, Getu Teferi

**Affiliations:** 1 Department of Sport Science, Debre Markos University, Debre Markos, Ethiopia

**Keywords:** blood pressure, percent body fat, cardiorespiratory fitness, aerobic exercise, resistance exercise, hypertension

## Abstract

**Background:**

Physical exercise is a well-established method for managing blood pressure (BP). The purpose of this study was to compare the effectiveness of aerobic, resistance, and a combination of both aerobic plus resistance training on BP, body composition (BC), and cardiorespiratory fitness (CRF) among hypertensive patients.

**Methods:**

The total population was sixty hypertensive patients; of these, forty-eight male adults (45.28 ± 7.44, years); with a sedentary lifestyle were randomized to one of the three exercise interventions or a control group. Subjects in all three exercise groups had an equal total exercise time (60 minutes), which included 10-15 minutes of warming up and dynamic stretching, 10-15 minutes of cooling down and static stretching and 30- 40 minutes for the main workout. Data were presented as mean (standard deviation, SD) or mean change with 95% confidence intervals (CI).

**Results:**

All three-intervention trainings showed improvement in BP, CRF, and BC. However, the most effective intervention training was combined training. Combined training resulted in significant reductions in body composition, resting heart rate (RHR) and, BP: body weight -7.92 kg, BML -5.96 kg/m2, SBP -17.75mmHg, DBP -12.5 mmHg, RHR -8.17 bpm, and percent body fat (%BF) -6.49%. The aerobic training group only increased VO2max 12.44 ml/kg/m.

**Conclusion:**

Compared to aerobic or resistance training alone, a 12-week of combination exercise may offer more comprehensive advantages for those at a higher risk for hypertension.

## Introduction

Chronic elevations in blood pressure (BP) are a hallmark of the multifactorial condition known as hypertension, which is strongly linked to adverse health outcomes(1). It is the most significant risk factor for mortality and increases the likelihood of cardiovascular disease (CVD) However, one of the most significant modifiable risk factors for cardiovascular disease prevention is hypertension (2). Hypertension (HTN) affects nearly 1.4 billion adults and is estimated to affect 30% of the world's population(3). By 2025, the World Health Organization (WHO) predicts that this number will rise to 1.6 billion(4). It is essential to improve cardiovascular disease risk factors. Physical exercise is a well-established method for controlling blood pressure in hypertensive patients(5) and recognized as a way of life changing, is the initial line of treatment recommended by many governing bodies (6). Most exercise recommendations concentrate on prescribing aerobic exercise (7). The health importance of aerobic exercise well established (8, 9). However, less knowledge exists with reference to the health impact of resistance exercise, particularly on people with elevated BP (10), further, different studies have showed that not utilizing combination exercise (resistance plus aerobic), most studies with an aerobic exercise alone(11, 12).

Recent meta-analyses and reviews have shown that aerobic and resistance training can both significantly lower blood pressure by 3–4 millimeters of mercury in both systolic blood pressure (SBP) and diastolic blood pressure (DBP)(13). However, most previous exercise treatment studies on BP and other CVD risk factors have primarily focused on aerobic or strength training alone. Few studies have reported the effects of strength exercise on cardiovascular disease (CVD), either alone or in conjunction with aerobic exercise. It is possible that resistance training and aerobic exercise produce distinct physiological responses. It would be beneficial to the general public as well as practitioners and their exercise prescriptions if the addition of strength exercise to aerobic exercise could have an additive effect and further reduce the risk of hypertension (14). The majority of previous studies on physical exercise and hypertension lacked random assignment or a control group and focused only on aerobic or resistance training (15). Additionally, healthy individuals were the primary subjects of previous research (15).

Aerobic and resistance training are typically the two main types of exercise training. Strength, power, and skeletal muscle mass can all be improved through resistance training(16). However, because the exercise intervention-mediated outcomes are inconsistent with various exercise intensities, age, and health conditions, the effects of resistance exercise training on blood pressure (BP), body composition, and cardiorespiratory fitness remain unclear. Aerobic exercise consistently enhances cardiopulmonary fitness and vascular health, in contrast to resistance training, by, for instance, increasing aerobic capacity and lowering blood pressure and arterial stiffness (17).

As a result, it is unclear whether concurrent aerobic and strength training would result in a better improvement in hypertensive patients' blood pressure, body composition, and cardiorespiratory fitness than either training only group. The purpose of this study was to compare the effectiveness of aerobic, resistance, and a combination of both aerobic plus resistance training on blood pressure, body composition, and cardiorespiratory fitness among hypertensive patients compared to a non-exercising control group.

## Materials and Methods

**Study setting, design, and sampling**: To achieve the objective of this study, from the total target population of sixty (60) male hypertensive patients, a total of forty-eight (45.28 ± 7.44 years) were selected based on the inclusion criteria. This study was conducted in Finoteselam Hospital, Jabi Tehinan Wereda, West Gojjam Zone, Ethiopia. The research design of this study was reflective of a “classic design for exploring cause-and-effect relationships”, the pretest-posttest parallel groups experimental design (18).

**Figure 1:**
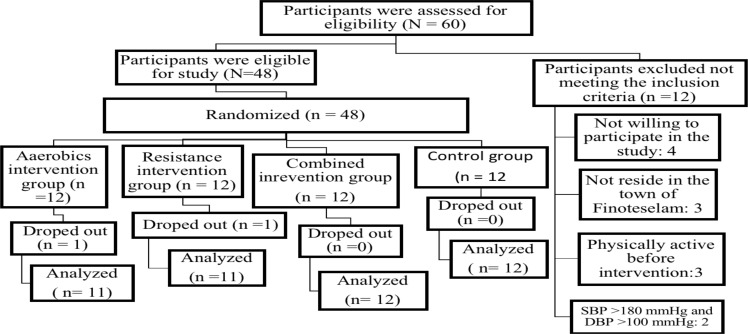
Participant flow chart

The study's source population consisted of all hypertensive patients who met the inclusion criteria. During the first two weeks prior to the study, hypertensive patients who visited the hospital were recruited. According to the hospital's general outpatient medical record, six to eight hypertensive patients visited the facility each day. If we use the lower limit, which covers five days per week, this equates to approximately 30 patients per week. Therefore, 60 hypertensive patients were considered the target population during the first two weeks prior to the study period, but 12 of them did not fulfill the inclusion criteria. The remaining 48 hypertensive patients were randomly divided into aerobic training group (ATG), resistance training group (RTG), combined training group (resistance plus aerobic; CTG) and control group (CG) (Figure 1).

With reference to the study of Ramirez-Campillo et al. (19), an a priori power analysis with an assumed Type I error of 0.05 and a Type II error rate of 0.20 (80% statistical power) was conducted for squat jump performance and revealed that 12 persons per group would be sufficient (20).

The baseline (pre-test) data were gathered prior to the intervention, and posttest data was gathered after the 12-week intervention. November 2022 through June 2022 comprised the study period.

**Inclusion and exclusion criteria**: **The** criteria for inclusion: 1) patients with hypertension aged 18 and above, 2) stage 1 and 2 hypertension patients who are willing to participate in the study (systolic blood pressure < 180 mmHg and diastolic blood pressure < 100 mmHg) (21), 3) residence in the town of Finoteselam (study area), 4) for at least the last three months before, patients were physically inactive and cleared a medical history form (the physical activity readiness questionnaire). Criteria for rejection: 1) People who are unfit for exercise and have hypertension-related complications, such as coronary heart disease, congestive heart failure, and cerebrovascular disease; 2) people who have other co-morbidities, such as diabetes mellitus, chronic kidney disease, and musculoskeletal injuries that prevent them from exercising.

**Data collection procedure and instruments**: The automated Sphygmacor XCEL (AtCor Medical, Itasca, IL, USA) was used to measure blood pressure and resting heart rate. The measurement of blood pressure and resting heart rate were carried out in the morning after emptying bladder but before taking blood pressure medication or drinking coffee, tea or taking meal (22). Additionally, should avoid potential stressors, such as checking work emails, watching the news, and doing exercise that may raise the blood pressure and resting heart rate (23).The participant was seated with their legs straight and a brachial pressure cuff placed over the brachial artery on their left arm. The device measured the brachial systolic and diastolic blood pressure three times, with a two-minute rest in between each measurement. For all measurements, the Sphygmacor XCEL reported the average of the last two readings instead of the initial reading.

Queens College Step Test was employed to measure cardiovascular endurance. The subjects were started by steps up and down on the platform at a given rate for three minutes. For three minutes, the subjects would begin by moving up and down on the platform at a predetermined rate. The recovery heart rate (HR) is then used to calculate the subject's Vo2max in mL kg-^1^min-^1^ using the following formula: Men:Vo2max = (mL kg^-1^ min^-1^) = 111.33 - (0.42.HR) (24).

Body mass index was calculated by dividing the height in meters squared by the body weight in kilograms using a standard stadiometer (Height & Weight Scale ZT-160 – NSL). Skinfold determination of percent body fat can be quite accurate when performed by a properly trained technician with a skinfold caliper (LeTkingok: High Precision Sebum Thickness Meter Skin Fold Caliper). It is estimated that the proportion of subcutaneous to total fat varies with gender and age. Gender and age are thought to have an impact on the ratio of subcutaneous fat to total fat(25). We used the Jackson-Pollock 3-Site Skinfold Formula for Body Density J-P 3-Site for men (26). As a result, the three-site formula for the chest, triceps, and subscapular is as follows: Body Density (BD) = 1.1125025 - 0.0013125 (sum of three skinfolds) + 0.0000055 (sum of three skinfolds) ^2^ - 0.000244(age). Body Density (BD) can be used to determine the percent of body fat (%BF), according to the Siri Equation (26); $\%\text{ BF}=\frac{495}{BD}-450$.

**Covariate variable**: A covariate is not part of the main research question, but could influence the dependent variable and therefore needs to be controlled for. Data collection instrument for measuring diet was food frequency questionnaire (27). Three-day dietary records for 2 weekdays and 1 day of weekend per week before intervention and after follow-up were taken.

**Exercise interventions**: During the 12-week training period, all study participants, with the exception of the non-exercise control group, exercised for 60 minutes, which included 10-15 minutes of warming up and dynamic stretching, 10-15 minutes of cooling down and static stretching (28), and 30- 40 minutes for the main workout. All training sessions were overseen by health fitness professionals and exercise physiologists. The American College of Sport Medicine's (29) served as the foundation for the exercise protocols. The total time spent on each session was used to equalize the exercise protocols. The aerobic exercise-only group performed aerobic dance at moderate intensity or an initial heart rate of 64% HRmax and gradually increased to approximately 76%HRmax as the intervention progressed (29). The heart rate monitor that was worn by the participants throughout each exercise session recorded their maximum heart rate, which could be exercised at an intensity that did not exceed 80% of their maximum heart rate. Additionally, until the intervention was completed, all subjects were instructed not to alter their eating habits or engage in additional moderate-to-vigorous physical activity.

The only resistance training (RT) group performed the following resistance exercises: standing plantar flexion, triceps pulley, neutral rowing, squatting, dumbbell supine, knee extension with ankle weights, dumbbell development, dumbbell curl, trunk flexion and, knee flexion with ankle weights (30). The circuit type of RT was used with intervals of 15–20s between exercises, with 3 sets of 10 repetitions with a rest of 1–2 minutes between sets. Due to the patients' lack of physical fitness and motor coordination, the loads were determined by their perceived exertion using the scale of 6 to 20 proposed by Borg in 1982 (31). The values used were 11 to 13, which represent a moderate effort.

For the combined (aerobic and resistance) training group, each session consisted of 30 minutes of resistance training and 30 minutes of aerobic exercise. The resistance training includes: knee extension with ankle weights, triceps pulley, squatting, dumbbell curl, and trunk flexion. These participants performed their resistance training with the same intensity and protocol as the aforementioned individual groups, except reducing it to five exercises instead of ten and two sets instead of three.

**Analysis**: All data were checked for normality through the Shapiro-Wilk test. Descriptive statistics was calculated for each variable and presented as a mean (standard deviation, SD) or mean change with 95% confidence intervals (CI). Paired t-test for comparing means of study variables within a group (pre-test and post-test) and one way of ANCOVA to control covariate variable (diet and age), determine their effect size, and to identify the most effective intervention group. During multiple comparison tests, we applied a Bonferroni's correction when the ANOVA results were significantly different. SPSS Statistics for Windows (version 24.0; Chicago, IL, USA: SPSS Inc) was used to conduct the analyses; P values were calculated, statistical significance was set at < 0.05.

**Ethical considerations**: The present study was carried out in accordance with the principles of the Declaration of Helsinki and after the approval of the Research Ethics Committee ((Ref. SPSC06/22). The researchers explained the objectives of the study to the participants, and informed consent was obtained from all participants.

## Results

Nighty six percent of participants were completed the exercise intervention. At baseline, participants mean age was 45.28 (7.44) years old; all were men and had a body mass index of 27.9 kg/m^2^ (1.89), SBP 154.11 (4.95) mmHg, DBP 92.96 (6.11) mmHg, resting heart rate 78.91 (3.67) bpm, vo2Max 28.83 (4.85) ml/kg/m and body fat percentage (%BF) 29.08 % (3.19). Demographic characteristics of subjects on baseline data revealed that there were no significant differences in study variables among the four randomized groups prior to intervention (Table 1).

**Table 1:** Baseline participant characteristics

	All	ATG	RTG	CTG	CG

N	46	11	11	12	12
Age, years	45.28 (7.44)	45.64 (6.58)	44.36 (7.83)	44 (7.97)	47.08 (7.88)
Height, meter	1.71 (.09)	1.72 (.09)	1.7 (.09)	1.71(.09)	1.71 (.08)
BW, kg	77.1 (5.06)	76.18 (4.45)	75.55 (5.94)	77.83 (6.44)	78.66 (2.57)
BMI, kg/m^2^	27.9 (1.89)	27.42 (1.845)	27.53 (1.86)	28.93 (1.94)	27.81 (1.76)
SBP, mmHg	154.11 (4.95)	154.09 (5.24)	154.09 (5.24)	154.16 9 (5)	154.08 (5.04)
DBP, mmHg	92.96 (6.11)	92.36 (5.64)	93.36 (6.36)	6.87 9 (93.5)	92.58 (6.23)
RHR, bpm	78.91 (3.67)	79.7273 (4.31)	79.45 (3.08)	79.08 (3.78)	77.5 (3.50)
Vo2Max, ml/kg/min	28.83 (4.85)	28.45 (5.05)	29 (5.08)	29.08 (4.99)	4.92 (28.75)
PBF, %	29.08 (3.19)	29.45 (4.48)	28.09 (2.51)	30.67 (.77)	28.06 (2.21)
Diet, calorie	2657.52 (115.09)	2647.73 (96.20)	2638 (98.55)	2666.75 (112.9)	2675.17 (152.57)

Data presented as mean (SD) for continuous variables. Abbreviations: ATG: aerobic training group, RTG: resistance training group, CTG: combined training group, CG: control group, BW: body weight, BMI: body mass index, SBP: systolic blood pressure, DBP: diastolic blood pressure, RHR: resting heart rate, PBF: percentage body fat.

All three training groups showed a significant improvement in blood pressure, body composition, and cardiorespiratory fitness from paired sample t-test (p < .05), but the control group did not show significant improvements (p> .05). However, combined training resulted in significant reductions in body composition and blood pressure: body weight -6.17 kg [95% CI: -4.07, -8.26], BMI -5.96 kg/m2 [95% CI: -3.79, -8.12], SBP -12.5 mmHg [95% CI: -15.52, -9.48], DBP -9.75 mmHg [95% CI: -12.54, -6.96], RHR -8 bpm [95% CI: -9.92, -6.08], and PBF -7.53%[95% CI: -10.31,-4.76]. The aerobic training group only increased VO2 max 9.36 ml/kg/m [95% CI: 5.48, 13.25] (Table 2).

**Table 2:** Mean differences between pretest and posttest of study variables through paired sample t-test

Variables and IGs	MD	SD	Std.Error	95% Confidence Interval		t	df	Sig.

				Lower	Upper			
**Aerobic training group**								
Body weight (BW), kg	-3.64	1.43	.43	-4.60	-2.67	-8.41	10.00	.00
BMI, kg/m^2^	-3.91	1.72	.52	-5.06	-2.75	-7.53	10.00	.00
SBP, mmHg	-9.36	4.88	1.47	-12.64	-6.08	-6.36	10.00	.00
DBP, mmHg	-6.64	3.35	1.01	-8.89	-4.38	-6.56	10.00	.00
RHR, bpm	-9.55	3.05	.92	-11.59	-7.50	-10.40	10.00	.00
Vo2max, ml/kg/m	9.36	5.78	1.74	5.48	13.25	5.37	10.00	.00
PBF, %	-5.62	3.95	1.19	-8.27	-2.97	-4.72	10.00	.00
Diet, calorie	-12.09	58.33	17.59	-51.28	27.10	-.69	10.00	.51
**Resistance training group**								
Body weight, kg	-3.27	1.42	.43	-4.23	-2.32	-7.64	10.00	.00
BMI, kg/m2	-2.71	.99	.30	-3.38	-2.05	-9.09	10.00	.00
SBP, mmHg	-6.91	4.83	1.46	-10.15	-3.67	-4.75	10.00	.00
DBP, mmHg	-7.55	4.34	1.31	-10.46	-4.63	-5.76	10.00	.00
RHR, bpm	-4.91	3.33	1.00	-7.15	-2.67	-4.89	10.00	.00
Vo2max, ml/kg/m	2.45	3.59	1.08	.04	4.86	2.27	10.00	.05
PBF, %	-1.91	2.17	.65	-3.36	-.45	-2.92	10.00	.02
Diet, calorie	-10.55	59.02	17.80	-50.20	29.11	-.59	10.00	.57
**Combined training group**								
Body weight, kg	-6.17	3.30	.95	-8.26	-4.07	-6.48	11.00	.00
BMI, kg/m2	-5.77	.94	.27	-6.36	-5.17	-21.30	11.00	.00
SBP, mmHg	-12.50	4.76	1.37	-15.52	-9.48	-9.10	11.00	.00
DBP, mmHg	-9.75	4.39	1.27	-12.54	-6.96	-7.69	11.00	.00
RHR, bpm	-8.00	3.02	.87	-9.92	-6.08	-9.19	11.00	.00
Vo2max, ml/kg/m	7.50	7.03	2.03	3.04	11.96	3.70	11.00	.00
PBF, %	-7.53	4.37	1.26	-10.31	-4.76	-5.97	11.00	.00
Diet, calorie	-11.08	55.73	16.09	-46.49	24.32	-.69	11.00	.51
**Control group**								
Body weight, kg	-.33	1.72	.50	-1.43	.76	-.67	11.00	.52
BMI, kg/m2	.40	.97	.28	-.22	1.02	1.43	11.00	.18
SBP, mmHg	.25	.87	.25	-.30	.80	1.00	11.00	.34
DBP, mmHg	-.08	.90	.26	-.66	.49	-.32	11.00	.75
RHR, bpm	-.83	1.53	.44	-1.80	.14	-1.89	11.00	.09
Vo2max, ml/kg/m	.08	.29	.08	-.10	.27	1.00	11.00	.34
PBF, %	-.11	1.71	.49	-1.20	.98	-.23	11.00	.83
Diet, calorie	74.58	140.2	40.47	-14.49	163.7	1.84	11.00	.09

Abbreviations: MD: mean difference, SD: standard deviation, df: degree of freedom, the mean difference is significant at p<0.01. Data presented as mean change (95% CI) for continuous variables

Combined training yielded the greatest benefit in body composition and blood pressure compared to the control group (Table 3), CTG vs. CG showed significant differences (p< .05) with reductions in BW -7 kg [95% CI: -2.92, -12.91], BMI -5.13 kg/m2 [95% CI: -2.73, -7.52], SBP -12.67 mmHg [95% CI: -6.22, -19.11], DBP -9 mmHg [95% CI: -2.36, -15.64], and PBF -4.82 % [95% CI: -2.17, -7.48]. However, cardiorespiratory fitness was improved in the aerobic training group; VO2max increased by 8.98 ml/kg/m [95% CI: 15.83, 2.14] and RHR reduced by -7.75 bpm [95% CI: -3.85, -11.65].

**Table 3:** Changes in body composition, blood pressure and cardiorespiratory fitness

Characteristics	Comparison groups	Mean change	SE	Sig.	95% Confidence Interval	
					**lower**	**upper**
Body weight (kg)	ATG vs. CG	-6.12*	1.84	.02	-.76	-11.49
	RTG vs. CG	-6.39*	1.84	.01	-1.03	-11.76
	CTG vs. CG	-7.00*	1.80	.00	-1.75	-12.25
BMI	ATG vs. CG	-4.78*	.84	.00	-2.33	-7.24
	RTG vs. CG	-3.47*	.84	.00	-1.02	-5.93
	CTG vs. CG	-5.13*	.82	.00	-2.73	-7.52
SBP	ATG vs. CG	-9.61*	2.26	.00	-3.01	-16.20
	RTG vs. CG	-7.15*	2.26	.03	-.56	-13.74
	CTG vs. CG	-12.67*	2.21	.00	-6.22	-19.11
DBP	ATG vs. CG	-7.02*	2.33	.04	-.23	-13.81
	RTG vs. CG	-6.93*	2.33	.04	-.14	-13.72
	CTG vs. CG	-9.00*	2.28	.00	-2.36	-15.64
RHR	ATG vs. CG	-7.75*	1.34	.00	-3.85	-11.65
	RTG vs. CG	-3.57	1.34	.08	-.33	-7.47
	CTG vs. CG	-6.67*	1.31	.00	-2.85	-10.48
Vo2max	ATG vs. CG	8.98*	2.35	.01	15.83	2.14
	RTG vs. CG	2.62	2.35	.74	9.46	4.22
	CTG vs. CG	7.75*	2.30	.02	14.44	1.06
Percent body fat	ATG vs. CG	-4.12*	.93	.00	-1.40	-6.83
	RTG vs. CG	-1.77	.93	.32	-.94	-4.49
	CTG vs. CG	-4.82*	.91	.00	-2.17	-7.48
Diet (calorie)	ATG vs. CG	-66.86	51.42	.64	-82.89	216.61
	RTG vs. CG	-75.05	51.42	.55	-74.70	224.80
	CTG vs. CG	-46.83	50.29	.83	-99.63	193.29

Abbreviations; BMI: Body mass index, SBP: Systolic blood pressure, DBP: Diastolic blood pressure, RHR: resting blood pressure, vs.: versus, SE: standard error, sig.: significant

ANCOVA was used to compare the effects of three different types of intervention training on body composition, BP, and Vo2 max whilst controlling for diet, and age, as shown in Table 4. There was a significant difference in the mean improvement of BW [F (3, 40) =8.01, p < 0.001], BMI [F (3, 40) = 14.07, p < 0.001], SBP [F (3, 40) = 10.78, p < 0.001], DBP [F (3, 40) = 5.40, p=0.003], Vo2Max [F (2, 40) = 6.01, p < 0.005], and PBF [F (3, 40) = 14.11, p < 0.001] between the exercise intervention groups whilst adjusting for diet and age. Furthermore, a comparison of the estimated marginal means revealed that combined training was more effective than aerobics or resistance training alone. The partial Eta Squared value indicates the effect size and should be compared with Cohen's guidelines (0.2 – small effect, 0.5 – moderate effect, 0.8 – large effect). It can be seen that for the exercise intervention effect size of BW (.38), BMI (.51), SBP (.45), DBP (.29), Vo2Max (.31), and %BF (.52). Additionally, the influence of diet on body composition, blood pressure, and cardiorespiratory fitness was small in the current study (Table 4).

**Table 4:** Effect size of diet, age, and exercise intervention on body composition, BP and Vo2Max

Variables and source	Type III Sum of Squares	df	Mean Square	F	Sig.	Partial Eta Squared
**Body weight**						
Corrected Model	480.17	5	96.03	5.34	.001	.40
Intercept	934.79	1	934.79	51.95	.000	.57
Calorie	96.97	1	96.97	5.39	.025	.12
Age	1.78	1	1.78	.099	.76	.00
Exercise intervention	432.629	3	144.21	8.01	.000	.38
Error	719.766	40	17.99			
**Body mass index**						
Corrected Model	199.97	5	39.99	9.62	.000	.55
Intercept	40.96	1	40.96	9.86	.003	.19
Calorie	.073	1	.073	.018	.89	.000
Age	4.73	1	4.73	1.14	.29	.03
Exercise intervention	175.42	3	58.47	14.07	.000	.51
Error	166.24	40	4.16			
**Percent body fat**						
Corrected Model	213.01	5	42.60	10.07	.000	.56
Intercept	140.43	1	140.43	33.19	.000	.45
Calorie	30.64	1	30.64	7.24	.010	.15
Age	9.74	1	9.74	2.30	.14	.054
Exercise intervention	179.06	3	59.68	14.11	.000	.514
Error	169.27	40	4.23			
**Systolic blood pressure**						
Corrected Model	1065.77	5	213.16	7.03	.000	.47
Intercept	1911.07	1	1911.07	63.01	.000	.61
Calorie	4.97	1	4.97	.164	.69	.004
Age	17.17	1	17.17	.57	.47	.014
Exercise intervention	981.16	3	327.05	10.78	.000	.45
Error	1213.206	40	30.330			
**Diastolic blood pressure**						
Corrected Model	598.92	5	119.78	3.78	.007	.32
Intercept	726.99	1	726.99	22.94	.000	.36
Calorie	11.04	1	11.04	.35	.56	.01
Age						
Exercise intervention	513.71	3	171.24	5.40	.003	.29
Error	1267.89	40	31.69			
**Vo2Max**						
Corrected Model	627.36	5	125.47	3.775	.007	.321
Intercept	118.05	1	118.05	3.55	.067	.08
Calorie	.78	1	.78	.023	.879	.001
Age	.795	1	.795	.024	.878	.001
Exercise intervention	599.021	3	199.674	6.01	.002	.311
Error	1329.357	40				

## Discussion

The primary objective of this study was to compare the improvements in blood pressure, cardiorespiratory fitness, and body composition as a result of combined aerobic and resistance training with that of the either training-only group. In this study, aerobic training group performed better than the combination and resistance group for cardiorespiratory fitness. The findings were consistent with the findings of a study by Church et al., (32).Their investigation revealed that, in comparison to resistance and combined training modalities, aerobic training significantly improved cardiorespiratory fitness.

In terms of blood pressure, the current study found that the combined intervention group showed more significant reductions in SBP and DPB compared to aerobic or resistances training alone. These findings are supported by literatures (33). Our results are consistent with those of previous studies, which showed that adult hypertensive patients' SBP and DBP more significantly decreased after 12 weeks of combined training. After a 12-week intervention, combined training (aerobic and resistance) showed more reduction in SBP and DBP compared to aerobic or resistance training alone (34) and combined (aerobic and resistance) exercise, which was recently demonstrated to be an efficient treatment for hypertension. Our findings are also supported by those of other studies that found significant improvements in either SBP or DBP with combination training (11). On the other hand, as can be seen in other studies that did not use combination exercise, most studies with participants in an aerobic-only group showed significant reductions in systolic blood pressure (11).

The use of combination exercise (aerobic plus resistance exercise) in research for blood pressure compared to aerobic or resistance exercise alone or a control group has had many different outcomes (33). These differences are often due to inconsistent methods, different populations, or a lack of appropriate stimulus to observe an effect (33).

Furthermore, the findings of some studies contradict with our investigation that blood pressure, body composition, and cardiorespiratory fitness changed significantly in the resistance-only group compared to the control group, but the changes were smaller than in the aerobic-only or a combination group(12). However, the resistance exercises-only group did not see as much improvement in blood pressure, with only a few studies reporting it.

Blood pressure, body composition and other CVD risk factors have previously been shown to be reduced by combined training (35). In the current study, all three training methods improved body composition, blood pressure and cardiorespiratory fitness, but the combined training group (aerobic plus resistance) resulted in the greatest improvements compared to the aerobic and resistance-only group. In line with our results, regular aerobic and resistance training both reduce total body fat, indicating that exercise training is a useful tool for healthy weight management (36). On other hand, a recent metaanalysis of randomized controlled trials comparing aerobic, resistance, and combination training found that aerobic training led to a greater reduction in body composition (body weight, BMI, and body fat percentage) than either combination or resistance training(33). These findings are in contradiction to the results obtained in this study.

Our findings indicated that all three training groups ATG, RTG, and CTG improved cardiorespiratory (resting heart rate); however, the aerobic training group led to a greater reduction in heart rate. The findings of this study were also supported by previous literatures (37). They came to the conclusion that patients' blood pressure, and heart rate improvements were statistically significant after six weeks of aerobic training (38). On the other hand, one study found that aerobic exercise had a significant impact on resting blood pressure and heart rate in prehypertensive and stage 1 hypertensive subjects and that after six weeks of aerobic training, these values significantly decreased in hypertensive individuals (39).

Generally, our findings revealed that blood pressure, body composition, and cardiorespiratory fitness were improved by all three intervention trainings, but the most promising intervention training was a combination of resistance plus aerobic training compared to resistance or aerobic training alone. However, additional research on the overall body composition, blood pressure, and cardiorespiratory fitness benefits of combinations of exercise for the general population and women is clearly required.
